# An exploratory study of predictors of cognition in two low-income samples of infants across the first year of life

**DOI:** 10.1371/journal.pone.0238507

**Published:** 2020-09-03

**Authors:** Viviane Valdes, Lara J. Pierce, Christianne Joy Lane, Emily B. Reilly, Sarah K. G. Jensen, Alma Gharib, Pat Levitt, Charles A. Nelson, Barbara L. Thompson

**Affiliations:** 1 Department of Pediatrics, Division of Developmental Medicine, Boston Children’s Hospital, Boston, Massachusetts, United States of America; 2 Harvard Medical School, Boston, Massachusetts, United States of America; 3 Department of Preventive Medicine, Keck School of Medicine, University of Southern California, Los Angeles, California, United States of America; 4 Department of Pediatrics, Children’s Hospital Los Angeles, Los Angeles, California, United States of America; 5 Viterbi School of Engineering, University of Southern California, Los Angeles, California, United States of America; 6 Program in Developmental Neuroscience and Neurogenetics, The Saban Research Institute, Children’s Hospital Los Angeles, Keck School of Medicine, University of Southern California, Los Angeles, California, United States of America; 7 Harvard Graduate School of Education, Cambridge, Massachusetts, United States of America; 8 Department of Pediatrics and Human Development, College of Human Medicine, Michigan State University, Grand Rapids, Michigan, United States of America; Pennsylvania State University, UNITED STATES

## Abstract

**Objective:**

In this exploratory longitudinal study we assessed cognitive development in a community sample of infants born into predominantly low-income families from two different urban sites, to identify family and community factors that may associate with outcomes by 1 year of age.

**Method:**

Infant-mother dyads (n = 109) were recruited in Boston and Los Angeles community pediatric practices. Infant cognition was measured using the Mullen Scales of Early Learning when the infant was aged 2, 6, 9, and 12 months. Longitudinal linear mixed effects modeling and linear regression models explored potential predictors of cognitive outcomes.

**Results:**

Cognitive scores were lower than the reference population mean at both 6 and 12 months. There were site differences in demographics and cognitive performance. Maternal education predicted expressive language in Boston, and speaking Spanish and lower rates of community poverty were associated with greater increases in overall cognition in Los Angeles.

**Conclusion:**

This exploratory study identified a number of drivers of child development that are both shared across cohorts and unique to specific community samples. Factors influencing heterogeneity within and across populations both may be important contributors to prevention and intervention in supporting healthy development among children.

## Introduction

Delays in early cognitive development have been associated with persistent challenges across the lifespan, including poor academic outcomes, difficulty securing employment, lower income, higher rates of teenage pregnancy, and an increased likelihood of engaging in illegal activities [1–3]. A variety of both heritable and non-heritable factors, singly and together, have been suggested to impact the development of cognitive functions [4]. Importantly, early life experiences are known to influence cognitive development, and early environmental factors such as family income, maternal education, and poverty can predict cognitive outcomes at the school years and beyond. This suggests that identifying risk and protective factors from the earliest stages of development could help inform strategies to prevent or mitigate lasting impacts of early adverse experiences [5–23].

The UK Millennium Cohort Study found that children born into poverty had diminished cognitive performance at 3 and 5 years of age, and by their seventh birthday, test scores of children living in low-income households were 20 percentile points lower than their higher-income counterparts [24]. In the United States, similar findings have been reported in multiple studies as early as 2 years of age and followed into adolescence [8, 15, 20, 22]. Children living below the poverty threshold and up to 1.5 times above the threshold, particularly those living persistently in poverty (greater than 4 years), scored 6 to 13 points lower on tests of verbal ability, IQ, and academic achievement [8, 15, 20, 22]. These patterns also were linked to school completion, in which among low-income children, a $10,000 increase in family income at age 5 was associated with a full year increase in school completion, and fewer behavioral and emotional problems [8, 11, 12, 23].

The effects of family and community poverty are complex factors that together have been challenging to resolve. It has been hypothesized that variation in factors such as proximity and access to high quality childcare, mental health care, healthy foods, recreational or green spaces, as well as exposure to violent crimes may all be associated with cognitive scores in early childhood [8, 9, 14, 25, 26]. Increased exposure to risk factors and insufficient or absent factors that reduce risk within a community may be one means by which elevated community poverty levels may negatively impact cognitive development, whereas the balance of specific risk and mitigative factors may point to mechanisms underlying these effects [27, 28].

Beyond the role of family and community poverty, maternal psychological factors have been explored as potential correlates of child development. Specifically, the association between maternal depression and infant cognitive abilities also has been examined as both a mediator and moderator of cognitive outcomes, albeit with varying results. Some studies indicate that increased prevalence of symptoms of maternal depression, pre- or postnatally, are negatively associated with cognitive development [29–34], however other studies report inconsistent relations [35, 36].

Additionally, social factors, such as primary language at home or care arrangements correlate with cognitive performance. For example, infants in bilingual households in the United States may display lower cognitive scores earlier in childhood, but they tend to outperform children in monolingual households on tasks of executive functioning later in development [37]. Similarly, there are benefits in both language skills and academic outcomes for children who maintain exposure to their first language while learning the majority language of the community [38]. Studies of care arrangements suggest that non-parental day care is associated with improved language scores in early childhood, though the effect was reduced over time [39]. Additional research suggests that non-parental care day care program may be protective for infants from low- but not high-income families [40].

Environmental factors influence early cognitive development, though the nature of these relations, particularly within low-resource contexts, is not well understood. Few studies have investigated the impact of experiential factors on early infant cognitive development [6, 17]. Because the foundations for cognition may be established during the earliest stages of development, when the brain is arguably most sensitive to the impact of certain experiences [41], understanding how environmental variables impact cognitive development in infancy is important for determining mechanisms underlying variation in developmental trajectories. In the present exploratory study, we draw upon the culmination of Early Life Stress (ELS) research [42, 43] and Experience Dependent Learning [44, 45] frameworks to investigate the developmental trajectory of cognitive scores and their potential association with specific environmental variables, in 2 to 12- month old infants living in primarily low-income resource households in two U.S. cities. To accomplish this, we: 1) recruited participants from two geographically distinct community pediatric practices to participate in a longitudinal study 2) characterized the developmental trajectory of cognitive scores in this cohort across the first year of life and 3) explored potential contextual risk factors (family and community demographics, maternal depression symptoms, and infant social factors) that may contribute to variation in infant cognitive scores.

## Materials and methods

### Participants

The current data were collected as part of a broader longitudinal study of 115 children conducted simultaneously in both Boston, Massachusetts and Los Angeles (LA), California. The study recruited infant-mother dyads from community pediatric practices affiliated with free-standing children’s hospitals. Participants were recruited at both sites following routine well-baby newborn and 1-month screenings. The inclusion criteria for the study were: receiving early postnatal services at the designated primary care clinics, mothers aged 18 or older with an infant who were < 2 months of age, and birth weight greater than the 20^th^ percentile (at least 2,500 grams). Medical assistants affiliated with the community pediatric practices approached mother-infant dyads that met inclusion criteria to determine interest in research participation. If mothers indicated interest, the medical assistant provided the research team with their contact information, and research personnel then contacted the families for further screening and possible enrollment in the study.

Exclusion criteria included infants who were born < 36 weeks of gestation; infants who had a known genetic, metabolic, or neurological disorder; infants who experienced birth complications (fetal distress, infant resuscitation, meconium respiration syndrome, *etc*.); infants who had identified congenital malformations or surgical interventions; or infants who had any severe sensory or motor impairments. Exclusion criteria were confirmed through patient medical records and pre-enrollment screening, though one infant born at 31 weeks was included due to inaccurate pre-enrollment screening. Study visits took place at 2, 6, 9, and 12 months from 2016 to 2019. The initial visit was conducted when the infant was between 2 months and 14 days and 3 months 14 days. The window for the 6, 9, and 12-month visits were +/- 4 weeks. Ethical approval for the study was obtained from Boston Children’s Hospital, IRB-P00019083, Research Network on Toxic Stress and Health and Children’s Hospital Los Angeles, CHLA-15-00267, Resiliency to Toxic Stress. Informed written consent was obtained from the mother for all infant-mother dyads who participated in the study. Participant compensation included pre-arranged ride share or travel reimbursement, meal vouchers, and monetary compensation.

### Measures

#### Cognitive scores

The primary outcome of interest was infant cognition measured using The Mullen Scales of Early Learning (MSEL) [46]. The MSEL is a standardized play-based developmental assessment that cross-cuts domains of gross motor, visual reception, fine motor, expressive language, and receptive language which are standardized to a t-distribution. An early learning composite score (ELC) is generated with four of the sub-scales (gross motor excluded per standard practice). This composite score is then standardized to compare cognitive developmental status from birth through sixty-eight months of age. The ELC uses a standardized score with a reference population mean of 100 and standard deviation of 15. Cognitive performance in LA was assessed at 2, 6, 9, and 12 months and in Boston at 6 and 12 months by trained members of the research team with advanced clinical, professional, and research degrees, in either English or Spanish based on the primary language spoken at home. The MSEL at the time of testing had not been adapted for Spanish-speaking individuals. However, there are very few verbal prompts in the MSEL from birth to 12 months. The MSEL has been routinely used to assess cognitive development, and was standardized using a sample of 1,849 children. The sample was considered to be representative of the United States (US) and included children aged 2 days through 69 months old. Participants in the sample were obtained from over 100 sites across the US. The sample was diverse and representative of census data in socioeconomic status, race/ethnicity, and sex.

Predictors of cognitive development included demographic variables and maternal depression symptoms gathered from the mother at study visits in her preferred language (Spanish or English). Three categories of variables of interest were created for analyses. These variables were: 1) family and community demographic factors, 2) maternal depression symptoms, and 3) infant social factors.

#### Family and community demographic factors

The family and community demographic factors were collected through self-report from mothers at the 2- month visit. Due to small representation of some categories, we combined data as follows. Marital status was represented as single (single, widowed, separated, divorced) or partnered (married, cohabiting). We collected maternal education in seven categories ranging from 8^th^ grade or less to M.D., Ph.D., J.D., or equivalent, which were combined into three categories: less than high school, high school or GED, and at least some college. The nine categories of annual family income ranging from <$5,000 to $100,000+ were stratified into <$16,000, $16,001–49,999, and $50,000+. As one-third of families did not disclose income, we derived neighborhood poverty levels as the percentage of households within participants’ neighborhoods below the federal poverty level from zip codes [47, 48].

#### Maternal depression

Maternal depression was assessed using the Edinburgh Postnatal Depression Scale (EPDS), a 10-item screening tool used to identify mothers at risk for perinatal depression [49–52]. The EPDS measures the experience of depressive symptoms over the past week. Scores range from 0 to 30, with possible clinical depression indicated by scores of 10 or greater. The EPDS was completed by the mothers at each research visit (2, 6, 9, and 12 months postpartum).

#### Infant social factors

The primary language spoken at home and infant childcare arrangements (parent or non-parent) were determined using a self-report response from the infant’s mother at each research visit. Language was dummy coded so that the individual effects of the two primary languages reported, English and Spanish, could be assessed.

### Statistical analysis

Data in this study were limited to participants who had at least one complete MSEL measurement in the infant (N = 109). Sample characteristics were first compared by site (Boston and LA). Pearson χ^2^ tests were performed for discrete categorical variables (marital status, maternal education, family income, primary language spoken in the house, childcare arrangements). Due to skewing in the data, EPDS scores were compared using a Mann-Whitney U test. *A priori* α to distinguish potential differences between sites was set as 0.05.

#### Developmental trajectory

To examine the developmental trajectory of MSEL ELC scores across the first year of life (2, 6, 9, and 12 months) within the LA sample, longitudinal linear mixed effects modeling was applied. The influence of the external environment including family and community demographics, maternal depression, and infant social factors reported at 2 months were included in the modeling. A diagonal covariance matrix was specified allowing observations within infants to be related while being independent from other infants. Age was explored as a non-linear variable in order to capture non-linear changes from 2 to 12 months, however there were considerable issues with fitting the Hessian matrix, so linear models were used. Age of the infant in months at each visit was used instead of study visit number, to take into account different time intervals between study visits across infants.

#### Predictors of change

To determine predictors of change in MSEL across the first year of life at both sites, linear regression models were used for analyzing the 6 to 12-month change, adjusting for 6-month scores. The influence of the external environment including family and community demographics, maternal depression, and infant social factors were included in the modeling. This modeling was performed with the sites combined and stratified by site. For the 2–12 month developmental trajectory analyses of MSEL for the LA site, participants were included if they had data from at least one time point (n = 56). For the combined 6–12 month predictor analyses, participants were included if they had MSEL data at both time point(s) and complete predictor data (n = 73). For both models (LA only and combined sites), baseline values of infant social factors were used, meaning for the 2–12 month trajectory for the LA site, the 2 month values were used, and for the 6–12 month trajectory for combined sites, the 6 month values were used.

While α = 0.05 was set as the *a priori* cutoff for statistical significance, we make note of α < 0.08 to indicate factors worth further review, given that this is an exploratory study with small sample size and, consequently, the power is low. Using G*Power, we estimated the detectible effect size for a difference score for our observed sample sizes in the models; with α = 0.05 and 80% power, we could detect an effect size (Cohen’s f^2^) of 0.11 or greater for the site combined model and 0.15 for the site stratified models. This translates into the proportion of variance accounted for by one of the predictors, accounting for all of the other predictors [53]. All analyses were performed using SPSS Version 25.0 (IBM Corp, Armonk, NY, USA).

## Results

### Sample characteristics

Six children had no MSEL measures due to missed research visits, yielding a total sample size for these analyses n = 109 (N_Boston_ = 53, N_LA_ = 56). The mean ELC score at 6 months was 89.79 (SD = 11.56; range 65–120); see Table 1. At 12 months, the mean ELC score was 94.81 (SD = 13.14; range 60–127).

**Table 1:** Infant MSEL scores.

		LA			Boston		
	Month	N	M	SD	N	M	SD
Early Learning Composite	2	54	84.04	9.05	0	.	.
	6	51	86.49	12.37	45	93.53	9.38
	9	44	88.18	13.35	0	.	.
	12	42	87.62	10.8	47	101.23	11.71
Gross Motor t-score	2	54	42.93	6.83	0	.	.
	6	51	40.90	9.59	44	50.77	7.95
	9	44	42.30	10.78	0	.	.
	12	42	40.38	9.21	46	49.13	11.51
Visual Reception t-score	2	54	41.41	10.09	0	.	.
	6	51	48.94	10.25	47	48.49	7.74
	9	44	47.20	9.86	0	.	.
	12	42	42.14	8.58	48	50.63	7.08
Fine Motor t-score	2	54	36.28	6.57	0	.	.
	6	51	40.80	10.02	47	48.13	5.72
	9	44	49.14	13.90	0	.	.
	12	42	49.43	9.47	48	56.27	9.47
Receptive Language t-score	2	54	41.24	7.04	0	.	.
	6	51	41.57	9.21	45	44.22	7.62
	9	44	33.91	7.84	0	.	.
	12	42	35.98	8.50	47	43.00	7.68
Expressive Language t-score	2	54	47.96	7.67	0	.	.
	6	51	39.45	8.59	46	46.22	7.28
	9	44	45.18	14.21	0	.	.
	12	42	46.69	8.41	48	51.85	7.19

MSEL Early Learning Composite Scores and sub-scale scores are presented for each site.

Abbreviations: LA, Los Angeles; M, Mean; SD, Standard Deviation

Overall sample characteristics are reported in Table 2. The populations represented at the Boston and LA sites were found to differ in several ways. Children from the Boston sample had a higher prevalence of single parenting compared to the LA sample (63% vs 43%, p = 0.045), mothers reported higher education levels (p = 0.009), higher income (p < 0.001), were more likely to speak English and less likely to speak Spanish (either monolingual Spanish or bilingual English and Spanish) (P’s < 0.001), scored higher on the EPDS at 6, 9, and 12 months (P’s < 0.005), and resided in neighborhoods with lower levels of community poverty (p < 0.001).

**Table 2:** Demographic information.

		LA		Boston		
**Family and Community Demographic Factors**		**N**	**%**	**N**	**%**	**p**
Marital Status	Single	23	43%	30	63%	0.045
	Partnered	31	57%	18	38%	
Maternal Education	Less than HS	15	27%	6	11%	0.009
	HS or GED	30	54%	23	43%	
	At least some college	11	20%	24	45%	
Family income	<$16,000	22	56%	8	21%	<0.001
	$16,000-$49,999	15	39%	16	42%	
	$50,000+	2	5%	14	37%	
		**N**	**M (SD)**	**N**	**M (SD)**	
Community poverty level*		56	28.4 (9.0)	50	19.3 (9.9)	<0.001
		**N**	**M (SD)**	**N**	**M (SD)**	
Maternal Depression (EPDS)*	2 Mo	56	4.1 (4.6)	50	5.0 (4.5)	0.358
	6 Mo	51	3.5 (4.0)	48	6.4 (5.0)	0.002
	9 Mo	44	2.8 (3.4)	47	5.5 (4.5)	0.002
	12 Mo	42	3.1 (3.5)	45	5.8 (4.6)	0.003
		**N**	**%**	**N**	**%**	**p**
% Depressed (EPDS ≥ 10)	2 Mo	8	14%	2	4%	0.066
	6 Mo	8	16%	13	27%	0.166
	9 Mo	5	11%	11	23%	0.132
	12 Mo	5	12%	8	18%	0.590
**Infant Social Factors**		**N**	**%**	**N**	**%**	**p**
Care Arrangement	Parent	36	64%	37	70%	0.540
	Non-Parent	20	36%	16	30%	
Primary Language Spoken in Home**	English	14	25%	41	79%	<0.001
	Spanish	27	48%	4	8%	<0.001
	Both	13	23%	1	2%	
	Other	2	4%	6	12%	
		**N**	**M (SD)**	**N**	**M (SD)**	
**Age of Infant at Visit**	2 Mo	54	2.5 (0.4)	59	2.3 (0.3)	
	6 Mo	51	6.1 (0.6)	45	6.4 (0.3)	
	9 Mo	44	9.1 (0.5)	50	9.4 (0.3)	
	12 Mo	42	11.8 (0.6)	47	12.5 (0.5)	

For each demographic variable, the number (N) and percentage (%) of participants are listed separately for each site. Where applicable, group Means (Standard Deviation) are presented. Pearson χ^2^ tests were performed for discrete categorical variables (marital status, maternal education, family income, primary language spoken in the house, childcare arrangements). Due to skewing in the data, EPDS scores were compared using a Mann-Whitney U test.

Abbreviations: EPDS, Edinburgh Postnatal Depression Scale; HS, High School; GED, General Education Diploma; LA, Los Angeles; M, Mean; Mo, Month; SD, Standard Deviation

* N = 106 for Community Poverty and EPDS due to 3 missing at Boston

**Spanish and English were treated as dummy variables, hence two statistical comparisons between sites.

### Developmental trajectory

**Fig 1:**
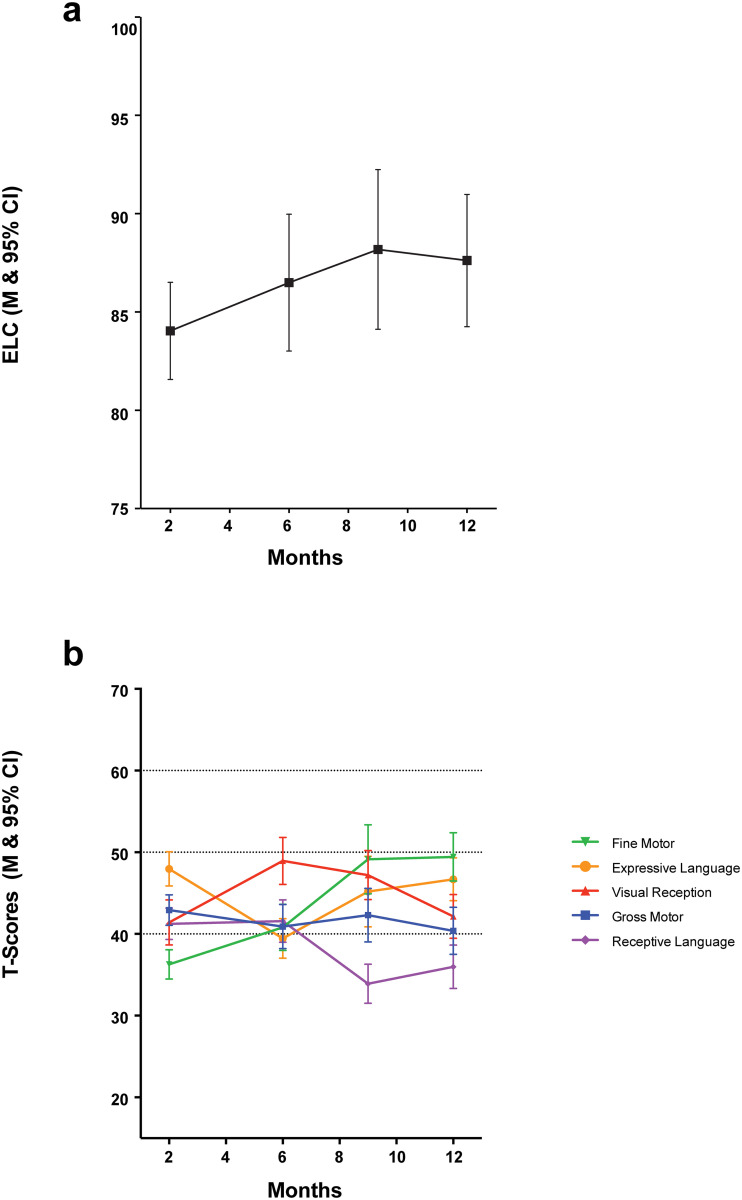
Developmental trajectory of MSEL ELC (a) and MSEL sub scale (b) scores across the first year of life for infants at LA (2 mo (n = 54); 6 mo (n = 51); 9 mo (n = 44); 12mo (n = 42)).

The pattern of MSEL scores for both the ELC and subscale scores for the LA site at 2, 6, 9, and 12 months are displayed in Fig 1. ELC scores are largely stable across time (Fig 1a), with small increases observed, on average, from 2 through 9 months before scores level out. Thus, there was not a significant effect of time (p = 0.94 in unadjusted model) on cognitive trajectories. When covariates of interest were added to the model, the linear time effect showed an increase of about 4 points on average from 2–12 months (β = 0.37, SE = 0.21) though statistically remained non-significant (p = 0.08). Poverty score also approached significance in this model (p = 0.056) with higher poverty being associated with a flatter ELC score trajectory.

Sub-scales of the MSEL showed variation in trajectory as the children developed across 2–12 months (Fig 1b). In the unadjusted model across time, there was a statistically significant time effect for Fine Motor and Receptive Language scores (P’s < 0.001), with Fine Motor performance significantly increasing an average of 14 points from 2–12 months (β = 1.38, SE = 0.16) and Receptive Language performance significantly decreasing on average 7 points from 2–12 months (β = -0.68, SE = 0.15). There were no linear time effects for Gross Motor (p = 1.0), Visual Reception (p = 0.71), or Expressive Language (p = 0.44) performance. This is possibly due to the linear modeling across time. In adjusted models, there was a significant effect of Spanish language (either monolingual Spanish or bilingual English and Spanish) (p = 0.03) spoken at home and community poverty levels (p = 0.04) on Fine Motor trajectories. Specifically, children immersed in a Spanish-speaking household had Fine Motor scores 4 points higher on average than those in homes without Spanish, and those families with higher poverty scores had infants with lower Fine Motor trajectories. In this adjusted model, the effect of time on Fine Motor trajectories was eradicated (p = 1.0), indicating that the predictors in the model explained the increases in fine motor skills. There was a trend (p = 0.06) towards concurrent maternal depression (EPDS) scores being associated with Gross Motor performance, with mothers who scored higher on the EPDS having infants who scored higher on Gross Motor skills; a 5 point increase in EPDS score was associated with an average increase of 1.5 in Gross Motor t-score. No other predictor had a statistically significant effect on subscale trajectories.

### Predictors of change

**Fig 2:**
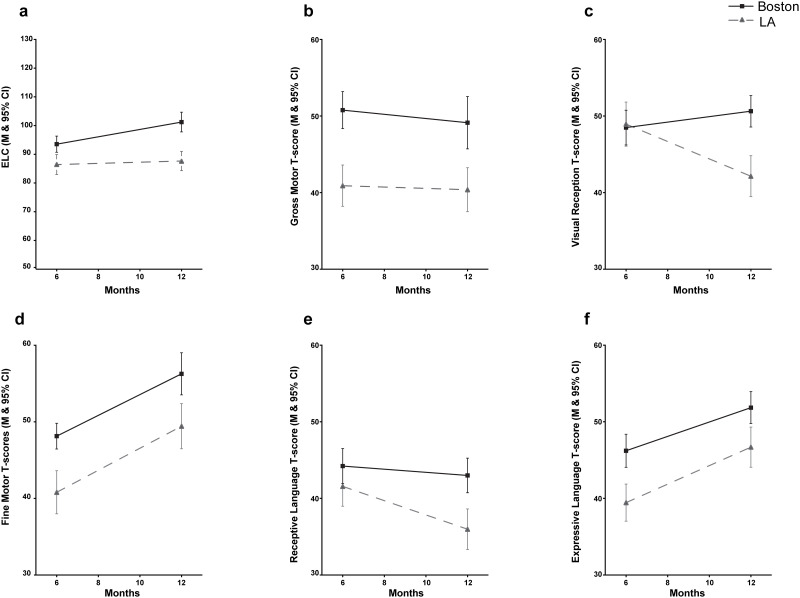
Differences in MSEL ELC (a) and sub-scale scores (b-f) between infants at Boston and LA at 6 and 12 months.

Fig 2 reports the ELC and subscale scores for the two sites at 6 and 12 months. There are several differences between sites in ELC and sub-scale scores with infants at the Boston site having consistently higher scores. The trends seen in the trajectory of the 6–12 month scores are typically in the same direction at both sites, with the exception of Visual Reception, which increased from 6–12 months at Boston and decreased at LA. The unadjusted values at 6 and 12 months, and the difference scores (12mo − 6mo) are presented in Table 3. Between 6 and 12 months there was a significant increase in the ELC (95% CI for ELC_Δ_ = 2.3, 8.4), which was driven by an increase at Boston, (95% CI for ELC_Δ_ = 4.0, 12.0), but not at LA (95% CI for ELC_Δ_ = -1.9, 7.4). Neither site showed a significant change in the Gross Motor sub-scale across time. Infants at LA exhibited a decrease in Visual Reception and Receptive Language sub-scale scores from 6 to 12 months, while those at Boston showed no significant change. Both sites showed significant increases in Fine Motor and Expressive Language sub-scales across time.

**Table 3:** Average infant MSEL scores for 6–12 months.

		Combined				Boston				LA			
		N	Mean	Lower CL	Upper CL	N	Mean	Lower CL	Upper CL	N	Mean	Lower CL	Upper CL
ELC	6M	96	89.79	87.45	92.13	45	93.53	90.71	96.35	51	86.49	83.01	89.97
	12M	89	94.81	92.04	97.58	47	101.23	97.8	104.67	42	87.62	84.25	90.98
	Diff 6-12M	80	5.36	2.33	8.39	39	8.1	4.2	12.01	41	2.76	-1.86	7.37
Gross Motor	6M t-score	95	45.47	43.41	47.53	44	50.77	48.36	53.19	51	40.9	38.21	43.6
	12M t-score	88	44.95	42.56	47.35	46	49.13	45.71	52.55	42	40.38	37.51	43.25
	Diff 6-12M t-score	80	-0.19	-2.41	2.03	39	-1.08	-4.42	2.27	41	0.66	-2.4	3.72
Visual Reception	6M t-score	98	48.72	46.9	50.55	47	48.49	46.22	50.76	51	48.94	46.06	51.82
	12M t-score	90	46.67	44.81	48.52	48	50.63	48.57	52.68	42	42.14	39.47	44.82
	Diff 6-12M t-score	83	-1.75	-4.29	0.79	42	2.21	-0.91	5.34	41	-5.8	-9.55	-2.06
Fine Motor	6M t-score	98	44.32	42.51	46.12	47	48.13	46.45	49.81	51	40.8	37.99	43.62
	12M t-score	90	53.08	50.98	55.18	48	56.27	53.52	59.02	42	49.43	46.48	52.38
	Diff 6-12M t-score	83	8.82	6.39	11.25	42	8.14	5.29	11	41	9.51	5.44	13.58
Receptive Language	6M t-score	96	42.81	41.08	44.55	45	44.22	41.93	46.51	51	41.57	38.98	44.16
	12M t-score	89	39.69	37.84	41.53	47	43	40.74	45.26	42	35.98	33.33	38.63
	Diff 6-12M t-score	80	-2.64	-4.89	-0.39	39	-1.13	-3.89	1.63	41	-4.07	-7.65	-0.49
Expressive Language	6M t-score	97	42.66	40.92	44.4	46	46.22	44.06	48.38	51	39.45	37.03	41.87
	12M t-score	90	49.44	47.73	51.15	48	51.85	49.77	53.94	42	46.69	44.07	49.31
	Diff 6-12M t-score	82	6.5	4.14	8.86	41	5.61	2.43	8.79	41	7.39	3.78	11

Unadjusted MSEL ELC and sub-scale values at 6 months, 12 months, and the difference score between 6 and 12 months for the combined sites and individual sites are presented.

Abbreviations: CL, Confidence Limit; Diff, Difference; ELC, Early Learning Composite; LA, Los Angeles; M, Month

#### Models of 6–12 month change

In the linear regression models of change from 6–12 months, the 6-month score was a consistent predictor of change. There were site differences for ELC which increased more at Boston (Mean ELC_Δ_ = 8.1, SD 12.1) than LA (Mean ELC_Δ_ = 2.8, SD 14.6, p<0.001), and a trend towards higher ELC scores in infants exposed to Spanish (either monolingual or bilingual English and Spanish) language in the home (p = 0.066) with infants exposed to Spanish having scores 7.4 points higher, on average (Table 4). Change in Fine Motor scores was similar to the change in overall score, with a site difference (Boston scores were 9.1 points higher, on average, p = 0.004), children exposed to Spanish had scores 9.6 points higher on average (p = 0.004), and community poverty was associated with a decrease in scores (p = 0.04). For Visual Reception and Receptive Language, there was a site difference, with Boston scoring 9.9 and 6.5 points higher, on average, respectively (P’s < 0.05). None of the other participant characteristics were significant in the combined model for Visual Reception and Receptive Language. There were no significant site differences in Expressive Language or Gross Motor scores, though both sites showed significant increases in Expressive Language across time (p < 0.05). None of the other participant characteristics were significantly associated with MSEL outcomes.

**Table 4:** Linear regression models of change from 6–12 months.

**Sites Combined**	**ELC**			**Gross Motor**			**Visual Reception**			**Fine Motor**			**Receptive Language**			**Expressive Language**		
	**B**	**SE**	**p**	**B**	**SE**	**p**	**B**	**SE**	**p**	**B**	**SE**	**p**	**B**	**SE**	**p**	**B**	**SE**	**p**
Boston	14.51	3.83	0.00	2.06	3.35	0.54	9.95	2.73	0.00	9.05	3.06	0.00	6.52	2.81	0.02	3.20	2.92	0.28
Single	-2.17	3.04	0.48	0.58	2.52	0.82	-0.86	2.15	0.69	-2.18	2.34	0.36	-2.19	2.28	0.34	2.00	2.24	0.38
<HS education	4.78	4.59	0.30	0.50	3.81	0.90	3.77	3.19	0.24	3.02	3.53	0.40	4.40	3.57	0.22	-2.93	3.25	0.37
HS education	-0.80	3.24	0.81	-2.13	2.77	0.45	-0.89	2.28	0.70	1.70	2.50	0.50	-0.27	2.45	0.91	-2.35	2.36	0.32
English Spoken in Home	2.51	3.51	0.48	4.18	2.90	0.16	1.16	2.53	-0.46	-0.65	2.76	0.11	0.02	2.58	1.00	-0.46	2.59	0.86
Spanish Spoken in Home	7.47	3.99	0.07	0.71	3.38	0.84	3.27	2.91	-1.13	-0.26	3.18	0.00	0.31	2.98	0.92	1.03	2.98	0.73
Mother primary child provider	-1.30	2.97	0.66	0.24	2.46	0.92	-1.77	2.11	0.84	-0.40	2.30	0.65	0.93	2.18	0.67	-0.18	2.16	0.94
Community Poverty Level	-0.21	0.15	0.17	-0.14	0.13	0.28	0.01	0.11	0.06	0.95	0.12	0.04	-0.16	0.11	0.17	-0.16	0.11	0.16
EPDS at 6 months	0.08	0.33	0.82	-0.01	0.28	0.97	0.20	0.24	0.83	0.41	0.26	0.67	-0.03	0.25	0.90	0.09	0.25	0.73
**Stratified by Site**																		
**Boston**	**B**	**SE**	**p**	**B**	**SE**	**p**	**B**	**SE**	**p**	**B**	**SE**	**p**	**B**	**SE**	**p**	**B**	**SE**	**p**
Single	-2.48	5.24	0.64	0.68	4.63	0.88	-1.29	3.12	0.68	-5.72	3.63	0.13	-0.81	3.85	0.84	-0.97	3.11	0.76
<HS education	4.79	7.89	0.55	7.74	7.56	0.32	4.42	4.86	0.37	9.14	5.37	0.10	5.79	5.88	0.33	-5.00	4.55	0.28
HS education	-9.62	6.68	0.16	-0.18	5.67	0.98	-5.49	3.65	0.14	-0.11	3.93	0.98	-3.64	4.34	0.41	-10.03	3.39	0.01
English Spoken in Home	6.56	6.24	0.30	5.56	5.35	0.31	1.82	4.06	0.66	4.61	4.34	0.30	-0.93	4.55	0.84	4.19	3.69	0.27
Spanish Spoken in Home	5.42	7.74	0.49	0.87	6.60	0.90	-0.91	5.02	0.86	6.74	5.48	0.23	-1.81	5.14	0.73	3.72	4.71	0.44
Mother primary child provider	2.70	5.47	0.63	4.61	4.52	0.32	0.82	3.21	0.80	4.99	3.68	0.19	0.69	3.70	0.85	0.34	3.12	0.92
Community Poverty Level	-0.16	0.26	0.54	-0.15	0.20	0.46	0.03	0.15	0.87	-0.27	0.17	0.13	-0.15	0.16	0.37	-0.09	0.14	0.51
EPDS at 6 months	0.77	0.60	0.21	0.29	0.45	0.52	0.63	0.33	0.07	0.27	0.39	0.49	0.25	0.39	0.53	0.50	0.32	0.13
**LA**	**B**	**SE**	**p**	**B**	**SE**	**p**	**B**	**SE**	**p**	**B**	**SE**	**p**	**B**	**SE**	**p**	**B**	**SE**	**p**
Single	1.14	3.88	0.77	0.25	3.30	0.94	0.46	3.12	0.88	1.12	3.37	0.74	-2.89	3.42	0.41	5.53	3.23	0.10
<HS education	3.26	6.02	0.59	-3.64	4.98	0.47	3.74	4.73	0.44	0.28	5.21	0.96	2.97	5.67	0.61	-1.22	4.85	0.80
HS education	5.15	4.22	0.23	-3.68	3.66	0.32	1.89	3.61	0.60	3.53	3.75	0.36	0.86	3.82	0.82	4.19	3.55	0.25
English Spoken in Home	1.86	4.50	0.68	2.74	3.87	0.48	1.78	3.80	0.64	4.08	3.97	0.31	0.59	3.97	0.88	-3.05	3.76	0.42
Spanish Spoken in Home	10.43	4.84	0.04	0.30	4.24	0.94	6.72	4.01	0.11	11.17	4.28	0.01	2.20	4.31	0.61	0.38	4.07	0.93
Mother primary child provider	-2.58	3.77	0.50	0.65	3.31	0.85	-3.23	3.08	0.30	-1.94	3.29	0.56	2.07	3.32	0.54	-0.54	3.14	0.86
Community Poverty Level	-0.51	0.23	0.04	-0.32	0.20	0.13	-0.28	0.19	0.17	-0.30	0.21	0.15	-0.31	0.21	0.15	-0.24	0.20	0.24
EPDS at 6 months	-0.06	0.55	0.92	-0.33	0.48	0.49	-0.08	0.45	0.86	-0.28	0.48	0.56	-0.25	0.49	0.61	0.21	0.46	0.66

Linear regression models were used for analyzing 6–12 month change in MSEL scores, adjusting for the 6-month scores. Modeling was performed with the sites combined and stratified by site.

Abbreviations: B, beta coefficient; ELC, Early Learning Composite; EPDS, Edinburgh Postnatal Depression Scale; HS, High School; LA, Los Angeles; M, Month; SE, Standard Error

#### Stratified by site

When stratified by site, Expressive Language had a statistically significant predictor at the Boston site; Education level was significantly predictive with scores of infants from mothers with at least some college being, on average, 10 points higher than those with a high school education and 5 points higher than those with less than high school education (p = 0.03). Additionally, there was a positive trend toward EPDS scores at 6 months predicting change in Visual Reception (p = 0.068). Within the LA site, speaking Spanish in the home and lower community poverty levels were associated with greater increases in ELC (P’s = 0.04) and Fine Motor skills (p = 0.01). None of the predictors were significantly associated with change in Visual Reception scores in LA.

## Discussion

The findings from our exploratory study provide additional support for the impact of early life experiences on cognitive development in the first year of life. This study specifically provides a descriptive inventory, at two different urban locations, of participating families and patterns of change in MSEL outcomes for infants across the first year of life. Infant cognitive scores on the MSEL from the combined sites were 10 points lower than the reference mean, on average, at 6 months, and 5 points lower on average, at 12 months. While the sample mean was still within the “average” range according to MSEL conventions at both 6 and 12 months, the sample mean ranked in the 22^nd^ percentile, indicating that many more of the participants in this study of early infancy fell “below average” of the reference group, (or within the 15^th^ percentile or lower at 6 months). At 12 months, the sample mean ranked in the 33^rd^ percentile. As additional points of reference regarding diverse populations, past research using the MSEL in Native American Indian communities, indicated that while at 6 months of age scores are near reference norms, a drop occurs between 6 and 15 months of age [54]. Similarly, past research in Gambia of early infancy did not find statistically significant differences in cognitive scores in the MSEL at 5–9 months (when compared to the reference population) but they did find significantly lower scores from 10–24 months [55], and a large study in Japan revealed delays beginning around 10 months [30]. Our findings indicate that within the Boston and LA samples, this drop may be occurring as early as 6 months.

### Developmental trajectory

Site-dependent developmental trajectories of ELC scores across the first year were also found. Cognitive scores at Boston increased notably from 6 to 12 months (mean score changed from 94 to 101), reaching the reference population mean on the MSEL. Unexpectedly and in contrast, the average scores at LA did not increase from 6 to 12 months (86 to 88). For both sites, the 12 month ELC was strongly predicted by 6 month ELC. These findings suggest that administration of the MSEL at 6 months, even during a routine pediatric visit, may provide an important opportunity, very early in development, to identify infants that would benefit from additional support and resources. The MSEL is relatively inexpensive for pediatric practices, can be administered at 6 months with a modest amount of training and practice by medical staff, typically in approximately 15 minutes, thus providing a scalable tool of cognition and learning for broad application [46].

Although ELC scores were stable over the first year of development, specific sub-scales did vary more over time. Infants in LA showed significant decreases in Visual Reception and Receptive Language from 6 to 12 months and infants at both sites showed significant increases in Fine Motor and Expressive Language across time. These findings suggest that examining different domains over time may provide valuable information to address individual differences that are not necessarily reflected in the overall ELC score. A note of caution however that typical development may be marked by uneven gains in modalities, so variation in slope of trajectory does not necessarily indicate a clinically significant lack of growth or pathology. Additionally, there are rapid biological changes that begin in the third trimester that extend through early childhood, such as myelination and cerebral cortical synaptogenesis that contribute to brain growth trajectories, potentially accounting for a portion of the variability in cognition within the first year of life [56–61].

### Predictors of change

#### Site scores

While substantive research indicates a relation between early family demographic factors and cognitive development [8, 9, 24, 25, 29, 35, 37–40], little is known about the emergence of effects in the first months of life. This study provides new information of the potential influence of these factors on cognition early in infancy, particularly within a high-risk context such as poverty. These findings may inform the development of interventions that can protect and bolster cognitive development well before pre-school onset, at a time when cognitive abilities predict academic success and delays can increasingly exacerbate the achievement gap often observed between children in low and high socioeconomic contexts. Recent findings note that in the United States, approximately 37% of children between the ages of 9 and 36 months receive any kind of developmental screening or surveillance in pediatric practices [62]. The results presented here reinforce the view that there is a massive need to implement scalable strategies for pediatricians to use for identification of early, atypical development.

#### Family and community demographics

In this study lower levels of community poverty were associated with higher ELC scores in infants at the LA site, and with higher Fine Motor scores across both sites. Maternal education also showed a site dependent impact, with infants of mothers who reported completing some college showing significant increases in expressive language sub-scale scores at Boston. These findings are not surprising in light of the existing body of research suggesting that adverse experiences such as family instability, lower levels of maternal education, and economic hardship are related to cognitive outcomes among school-aged children [8, 18, 24, 63, 64]. Our findings add to the existing literature by providing evidence for these observed effects in an already low-income sample within the first year of life.

#### Maternal depression

Surprisingly, in the Los Angeles cohort, there was a limited impact of higher maternal EPDS scores on cognitive performance associated with greater increases in ELC. This should be interpreted with caution, however, because we did not deliberately recruit or enroll women with increased risk for maternal clinical depression. Thus, in this low-risk population, scoring higher on the EPDS, but within the normal range, may not translate into clinical depression, but rather, reflect depressive symptoms, in contrast to prior findings indicating a negative impact of maternal clinical depression on infant cognition [65]. It should be noted that screening instruments and not structured clinical interviews were performed in this study.

#### Infant social factors

Spanish spoken at home, whether alone (monolingual) or with English (bilingual) was a positive predictor of ELC and Fine Motor sub-scale scores. This is consistent with findings of a cognitive or academic advantage for bilingual language learners, particularly those who maintain their heritage language. A bilingual advantage also has been observed from as early as 6–7 months of age using tasks of basic information processing [66] and cognitive control [67]. Additionally, we found no significant impact on infant cognitive scores based on the child’s caregiver (parent or non-parent) during the day.

### Site differences

Notably, while the study used the same inclusion and exclusion criteria and recruitment strategies in community pediatric practices, the enrolled families at each site differed in a number of ways, representing the distinct population diversity of each region. Specifically, based on the 2018 census, 20.5% of households in Boston and 14.9% in LA were below the federal poverty line and more than twice the proportion (44.9%) of the population in LA considered themselves Hispanic or Latinx compared to Boston [47]. While differences in MSEL administrators between the two sites is a potential confound, we believe this is unlikely as administration of the MSEL was performed by trained research personnel with advanced clinical, professional, and research degrees. Additionally, the 6-month MSEL data was not statistically different between sites. We did observe different associations at each site between environmental variables and MSEL outcomes, both for the ELC score and individual subscales. This heterogeneity indicates that for early developmental assessments, unique variances of each population must be taken into consideration when interpreting associations between environmental exposures and cognitive outcomes. These different associations suggest that cognitive development might exhibit differential sensitivity to environmental challenges in different populations of children, depending on the context in which they reside. These results lend support to findings that there are both common and unique drivers of child development, and identifying heterogeneity that drives these unique influences may be more relevant than generalizing across populations [68–72]. Considering heterogeneity in this way supports an intervention approach that would allow for more personalized interventions aimed at improving cognition and the overall health of the child. In other words, family and community demographics as well as social factors in the infant’s environment may generally be important predictors of change across sites. However, there were some site differences in regard to which specific exposures were linked to various developmental domains, as well as the strength of associations between these variables, that should be accounted for in the creation of community-adapted interventions. These universal challenges to early development may each exhibit differences in impacting the developing child, attributed to timing of the variable exposure, and to the differential sensitivity of each child [45].

### Future directions

Importantly, in this exploratory study, we were successful in setting up and collaborating with community pediatric clinics serving sociodemographic and culturally diverse populations, and believe this to be promising for future studies that seek to either expand on the existing population or engage families at other sites. Either can be achieved through careful protocol designs that facilitate utilization of pediatric practices and the populations that they serve. We implemented many steps to reduce the bias of recruiting through community pediatric practices, though the sample enrolled may still differ from that enrolled directly from the community at-large. Though successful in enrolling participants in this longitudinal study across two urban community clinics, the sample sizes are considered small. Additionally, lost data points due to missed visits or drop-out from the study also impacted statistical power, although retention across time points was high. Despite this limitation, our results demonstrate the importance of collecting larger samples from more diverse populations.

Other limitations within our sample are acknowledged (e.g., stratified sample sizes resulted in reduced power that may miss small within-site associations, LA recruited more families within the lower end of the socioeconomic range, fewer ages sampled in Boston), but it is important to consider region-specific differences that could account for the distinct associations observed across sites. For example, the distribution of neighborhoods with relatively high and low levels of poverty might differ across cities, resulting in different degrees of mobility between neighborhoods and influencing access to resources for different community groups. The present data do not directly address questions such as these but do highlight the importance of examining contextual factors within different geographic regions to better understand how specific variables are related to child cognitive outcomes. Additionally, beyond the variables included in the current study, other factors associated with poverty such as food insecurity, infectious disease, and other forms of psychosocial distress may also affect developmental trajectories [13]. Future research should aim to include measures of these variables to better understand their role in early development.

The current study identifies specific risk and protective factors that may contribute to cognitive development very early in an infant’s life. Providing opportunities for achieving higher levels of maternal education and reduced community poverty, ultimately optimizing home environments, may serve as relevant protective factors for infant cognitive development [64]. The first year of life includes sensitive periods of neurodevelopment during which foundational abilities are established [41], suggesting that there may be enduring consequences as a result of early experiences (positive or negative). Continued longitudinal follow-up of children in the current exploratory study, as they reach early childhood, provide an opportunity to examine the effect of experiences that occur at different points on developmental trajectories, allowing for further testing of these hypotheses. Additionally, we highlight the need to continue to incorporate into future research studies infants who are in higher risk situations (*i*.*e*., those with high levels of poverty plus high perinatal depression) to provide additional evidence supporting the findings of the current study.

This study contributes novel data to the growing literature by demonstrating the impact of early life experiences on cognitive development in the first year of life. The findings highlight the importance of the detailed analyses of environmental factors including education, maternal mental health, and language, beyond common analyses that treat SES as a single exposure. These findings reflect that both shared and unique drivers are important for cognitive development. The study further provides a framework for intervention efforts that support and improve healthy development of all children.

## Supporting information

S1 Data (XLSX)

## Abbreviations

ELC: Early Learning Composite
EPDS: Edinburgh Postnatal Depression Scale
GED: General Education Development
HS: High School
JD: Juris Doctor
LA: Los Angeles
MD: Doctor of Medicine
MSEL: Mullen Scales of Early Learning
PhD: Doctor of Philosophy
SES: Socioeconomic Status
UK: United Kingdom
